# Management of sepsis in neutropenic patients: 2014 updated guidelines from the Infectious Diseases Working Party of the German Society of Hematology and Medical Oncology (AGIHO)

**DOI:** 10.1007/s00277-014-2086-0

**Published:** 2014-04-29

**Authors:** Olaf Penack, Carolin Becker, Dieter Buchheidt, Maximilian Christopeit, Michael Kiehl, Marie von Lilienfeld-Toal, Marcus Hentrich, Marc Reinwald, Hans Salwender, Enrico Schalk, Martin Schmidt-Hieber, Thomas Weber, Helmut Ostermann

**Affiliations:** 1 Department of Hematology, Oncology and Tumourimmunology, Charité Campus Virchow Klinikum, Augustenburger Platz 1, 13353 Berlin, Germany; 2 Department of Oncology, Hematology and Stem Cell Transplantation, Medical Faculty, RWTH Aachen University, Aachen, Germany; 3 3rd Department of Internal Medicine, University Medical Centre Mannheim, University of Heidelberg, Heidelberg, Germany; 4 Internal Medicine IV, Oncology and Hematology, University Hospital of Martin-Luther-University, Halle (Saale), Germany; 5 Department of Hematology and Oncology, Klinikum Frankfurt Oder, Frankfurt, Germany; 6 Department of Hematology and Oncology, University of Jena, Jena, Germany; 7 Department of Hematology, Oncology and Palliative Care, Harlaching Hospital, Munich, Germany; 8 Department of Hematology and Oncology, Asklepios Hospital Altona, Hamburg, Germany; 9 Department of Hematology and Oncology, Medical Centre, Otto-von-Guericke University Magdeburg, Magdeburg, Germany; 10 Clinic for Hematology, Oncology and Tumourimmunology, HELIOS Clinic Berlin-Buch, Berlin, Germany; 11 3rd Department of Internal Medicine, Ludwig-Maximilians University, Munich, Germany

**Keywords:** Guideline, Sepsis, Neutropenia, Management

## Abstract

Sepsis is a major cause of mortality during the neutropenic phase after intensive cytotoxic therapies for malignancies. Improved management of sepsis during neutropenia may reduce the mortality of cancer therapies. Clinical guidelines on sepsis treatment have been published by others. However, optimal management may differ between neutropenic and non-neutropenic patients. Our aim is to give evidence-based recommendations for haematologist, oncologists and intensive care physicians on how to manage adult patients with neutropenia and sepsis.

## Clinical significance and methods

Sepsis is a leading cause of mortality in patients with haematologic malignancies or solid tumours undergoing intensive cytotoxic chemotherapy [29, 168]. Therefore, optimization of diagnosis and management of sepsis could improve outcome of intensive cytotoxic therapies. A number of prior guidelines on the management of sepsis have been published [15, 41, 43, 60, 96, 106, 132, 137]; however, none of these guidelines specifically address diagnosis and management of sepsis in neutropenic patients.

These updated guidelines were written to provide guidance on diagnosis and management of sepsis in the neutropenic host. First, a panel of 13 experts in the field of infectious diseases in haematology and oncology agreed to participate in preparing the guidelines. Second, the guidelines were thematically divided into six subtopics. Then, subcommittees of two to four authors were created, being responsible for literature search in one of the subtopics. We systematically searched Medline for English language publications up to June 2013 using the key terms: sepsis and one of the following: neutropenia, bacteraemia, bloodstream infection (bacteraemia), definition, epidemiology, incidence, risk factors, prognosis, treatment, antibiotic, antifungal, cardiovascular, pulmonary failure, ventilation, renal dysfunction, renal failure, dialysis, haemofiltration, nutrition, hyperglycaemia, steroid, coagulation, growth factor, immunoglobulin and transfusion. Meeting abstracts were not included; however, references generated from published guidelines and reviews were also investigated. The consensus process was performed as an email- and meeting-based discussion group. In a second step, the manuscript draft was peer reviewed by the review committee of the Infectious Diseases Working Party of the German Society of Hematology and Medical Oncology (AGIHO) on October 1st, 2013. In a third step, the guidelines were approved by the assembly of the members on October 20th, 2013. Criteria used to quote levels and grades of evidence are as outlined in Table 1 [88]. The first draft of the manuscript was written by the subcommittees. The final version of the manuscript was prepared by the corresponding author and has been approved by all authors.

**Table 1:** Categories of evidence used in this guideline [88]

Category, grade	Definition
Strength of recommendation	
A	Good evidence to support a recommendation for use
B	Moderate evidence to support a recommendation for use
C	Poor evidence to support a recommendation
D	Moderate evidence to support a recommendation against use
E	Good evidence to support a recommendation against use
Quality of evidence	
I	Evidence from ≥1 properly randomized, controlled trial
II	Evidence from ≥1 well-designed clinical trial, without randomization; from cohort or case-controlled analytic studies (preferably from >1 centre); from multiple time-series; or from dramatic results from uncontrolled experiments
III	Evidence from opinions of respected authorities, based on clinical experience, descriptive studies or reports of expert committees

## Definitions

A formal definition of sepsis has long been tried by several researchers and must lack specificity given the broad spectrum of reactions to pathogens.

We suggest using the diagnostic consensus criteria for sepsis adapted to neutropenic patients (Table 2) [97, 98]. In neutropenic patients, the white blood cell count cannot be used as a criterion to define sepsis. The definitions of severe sepsis and septic shock remain unchanged and refer to sepsis-induced organ dysfunction (Table 3).

**Table 2:** Diagnostic criteria for sepsis during neutropenia [97, 98]. In neutropenic patients, cytopenia cannot be used as a criterion to define sepsis

General parameters
Fever (core temperature >38.3 °C)
Hypothermia (core temperature <36.0 °C)
Heart rate (>90 bpm or >2 SD above the normal value for age)
Tachypnoea (>30 bpm)
Altered mental status
Significant edema or positive fluid balance (>20 mL/kg over 24 h)
Hyperglycaemia (plasma glucose >110 mg/dL or >7.7 mmol/L) in the absence of diabetes
Inflammatory parameters
Plasma C reactive protein or plasma procalcitonin (>2 SD above the normal value)
Haemodynamic parameters
Arterial hypotension (systolic blood pressure <90 mmHg, mean arterial pressure <70 mmHg, or a systolic blood pressure decrease >40 mmHg in or <2 SD below normal for age)
Mixed venous oxygen saturation (>70 %)
Cardiac index (>3.5 L/min/m^2^)
Organ dysfunction parameters
Arterial hypoxaemia (PaO2/FIO2 <300)
Acute oliguria (urine output <0.5 mL/kg/h for ≥2 h)
Creatinine increase (≥0.5 mg/dL)
Coagulation abnormalities (international normalized ratio >1.5 or activated partial thromboplastin time >60 s)
Ileus (absent bowel sounds)
Hyperbilirubinemia (plasma total bilirubin >4 mg/dL or 70 mmol/L)
Tissue perfusion parameters
Hyperlactataemia (>3 mmol/L)
Decreased capillary refill or mottling

**Table 3:** Definitions of severe sepsis and septic shock

Severe sepsis	Sepsis with new signs of organ dysfunction or a decrease in organ perfusion (lactate acidosis, oliguria (<30 mL/h or <0.5 mL/kg/h), hypotension (<90 mmHg or decrease of >40 mmHg), mental alteration)
Septic shock	Severe sepsis and hypotension persistent despite adequate fluid substitution and exclusion for other reasons for hypotension

## Incidence

Systematic data evaluating the overall incidence of neutropenic sepsis in cancer patients are lacking. The incidence of febrile neutropenia and bacteraemia has been studied more in detail, albeit the majority of studies did not use uniform definitions, include at least partly non-neutropenic patients and focus on distinct patient subgroups. Patients with solid tumours develop febrile neutropenia in around 10–40 %, but this complication might occur in more than 80 % of patients with haematological malignancies [1, 54]. In patients with indwelling central venous catheters (CVC), febrile neutropenia is frequently caused by catheter-related or catheter-associated bacteraemia with an incidence of around 10–20/1,000 neutropenic days [16, 34]. Likewise, translocation of gut organisms, such as vancomycin-resistant enterococci (VRE), may cause bacteraemia and, ultimately, sepsis in neutropenic cancer patients in up to 40 % of colonized patients [30, 101, 154].

It can be assumed that >50 % of patients with febrile neutropenia or bacteraemia develop sepsis using the consensus definition. Severe sepsis and septic shock, which have been investigated in a few prospective and retrospective analyses, might occur in up to 20–30 and 5–10 % of patients with febrile neutropenia, respectively [6, 78, 81, 95, 109, 112].

The increasing numbers of elderly patients undergoing intensive treatment modalities and patients infected with treatment-resistant organisms led to the assumption that the frequency of neutropenic sepsis will increase [11].

## Risk factors and prognosis

Prospective data of risk factors and prognosis for sepsis in adult neutropenic patients are rare [6, 63, 78].

### Risk factors for bacteraemia

There are few data on risk factors for bacteraemia during neutropenia. Apostolopoulou et al. observed a significant higher rate of bacteraemia, potentially resulting in sepsis, in patients with neutropenia <0.5 g/L. Additionally, acute myeloid leukaemia, a prolonged hospital stay, a Hickman catheter, or pre-treatment with antibiotics, chemotherapy or surgery were significantly associated with BSI in haematological and oncological patients [16].

### Risk factors for development of severe sepsis

The presence of hypophosphataemia (<0.8 mmol/L) and hypoproteinaemia (<62 g/L) have been identified as risk factors for severe sepsis in febrile neutropenia [78]. The development of septic shock in febrile neutropenia is independently predicted by the presence of pulmonary infection, tachypnoea, increased serum levels of procalcitonin (≥1.5 ng/mL), high lactate levels [6, 109], decreased serum levels of bicarbonate (<17 mmol/L), antithrombin (<70 %) or factor VIIa (<0.8 ng/mL) [63, 78, 109, 135]. A low Multinational Association for Supportive Care in Cancer (MASCC) risk index score of <21 is associated with an increased risk for septic shock in febrile neutropenic patients [6].

### Prognosis

Prolonged neutropenia <0.5 g/L or the delayed initiation of antibiotics is associated with poor clinical outcome in neutropenic patients with sepsis [5, 103]. Severe sepsis and septic shock negatively influence outcome [64, 95, 162]. A recent prospective study demonstrated mortality rates of 35 % in severe sepsis, 47 % in septic shock and 85 % in multi-organ failure in patients with haematological malignancies [19]. Factors that were significantly associated with hospital survival included remission of malignant tumour and time to intensive care unit (ICU) admission <24 h. Negative predictive factors for hospital survival were as follows: allogeneic haemopoietic stem cell transplantation (HSCT), poor performance status, invasive pulmonary aspergillosis, malignant organ infiltration and acute respiratory failure. In another study, Legrand et al. identified prognostic factors for neutropenic patients with severe sepsis or septic shock: The appearance of acute non-infectious complications, of neurological, respiratory or hepatic dysfunction, the need for vasopressor therapy, or older age increased the mortality. On the other hand, the early removal of a CVC and combined antibiotic therapy were associated with higher survival [95].

## Microbiology

Blood cultures as part of the usual microbiological work-up as per local protocol (including urine cultures, stool cultures etc.) remain the gold standard for the diagnosis of bacteraemia and fungaemia. Blood cultures should be standardized in terms of volume, culture sets, frequency, processing, interpretation and reporting.

However, although most episodes of febrile neutropenia are assumed to be caused by an infection, blood cultures are positive in less than 30 % of febrile neutropenic episodes [48]. Typical organisms causing sepsis during neutropenia are summarized in Table 4.

**Table 4:** Typical pathogens during bacterial sepsis in neutropenic patients

Origin	Frequent pathogens
Unknown	Coagulase-negative *Staphylococci*, *Escherichia coli*, *Enterococcus* species
Lung	*Pseudomonas aeruginosa*, *Streptococcus pneumoniae* (pneumococci), *Viridans* (alpha-haemolytic) *streptococci*, *Acinetobacter species*
Abdomen	*Escherichia coli*, *P. aeruginosa*, *Clostridium spp.*, *Enterococcus spp.*, *Klebsiella* species
Urogenital	*Escherichia coli*, *Klebsiella* species, *Pseudomonas aeruginosa*
Soft tissue	*Staphylococcus aureus*, alpha-haemolytic *streptococci*
CVC	Coagulase-negative *Staphylococci*, *Coryneform bacteria*, *Propionibacterium* species, *Candida albicans*, *Candida tropicalis*, *Candida parapsilosis*, *Stenotrophomonas maltophilia*

*CVC* central venous catheter

The epidemiology of gram-positive versus gram-negative bacteraemia varies in different countries [115]. In contrast to other countries, Germany has a predominance (>50 %) of gram-positive bacteria as the cause for febrile neutropenia [45]. Knowledge about the local epidemiology is essential for a rational choice of empirical antibiotic therapy as already pointed out by others. This is particularly true for colonization with resistant bacteria, since these have been associated with an increased risk of bacteraemia with these pathogens [101].

PCR-based methods to detect bacterial and fungal DNA have yet to be validated in larger cohorts [8, 93, 105, 120, 164]. In contrast, PCR-based methods play a definitive role in the diagnosis of viral infections, which may cause sepsis in severely immunocompromised patients [71, 102, 129].

## Treatment

### Antimicrobial treatment

Empirical antimicrobial treatment using broad-spectrum antibiotics must be started immediately in neutropenic patients with sepsis (AII). A large retrospective study including more than 2,000 patients showed that during severe sepsis, effective antimicrobial administration within the first hour of documented hypotension is associated with increased survival [95]. In this study, each hour of delay in antimicrobial administration over the ensuing 6 h was associated with an average decrease in survival of 7.6 % [95].

In neutropenic patients with sepsis, results from randomized controlled trials are lacking, and recommendations are based on study results from non-neutropenic patients as well as on expert opinions. We recommend initial treatment with meropenem or with imipenem/cilastatin or with piperacillin/tazobactam (AIII).

Meta-analyses show that a combination treatment with aminoglycosides increased renal toxicity without improving efficacy in neutropenic patients with bacteraemia [125–127]. However, in a retrospective study, the use of β-lactam antibiotic/aminoglycoside combinations were associated with superior outcome, as compared with single-agent antimicrobial treatment, in neutropenic patients with severe sepsis and septic shock [95]. Another retrospective study showed reduced hospital mortality in non-neutropenic patients with severe bacterial sepsis after combination therapy comprising at least two antibiotics of different mechanisms versus antibiotic monotherapy [92]. Taken together, a combination treatment with an aminoglycoside may be considered in neutropenic patients with septic shock and severe sepsis (BIII).

Knowledge of local microbiology data is crucial for the choice of antimicrobial agents. Importantly, local resistance patterns as well as colonization with resistant bacteria have to be considered [101]. If infection due to bacteria with frequent resistance to carbapenems or piperacillin/tazobactam is suspected, a specific antibiotic should be added (BIII). If a specific organ infection is suspected, antibiotic therapy should be adapted accordingly. Recommendations on antifungal therapy during neutropenia were recently published by our group and by others [55, 104, 118, 157].

### Treatment of cardiovascular insufficiency

Aggressive and early goal-directed treatment aiming at restoration of cardiovascular function is crucial [42, 140]. To restore adequate cardiac filling pressures and to maintain adequate organ perfusion (goal, mean arterial pressure 65 mmHg, central venous pressure 8–12 mmHg, pulmonary wedge pressure 12–15 mmHg, urinary output 0.5 mL/kg/h and central venous or mixed venous oxygen saturation 70 %), crystalloid fluids are recommended as the initial fluid of choice in severe sepsis and septic shock. Compared to crystalloids, randomized controlled trials did not show beneficial effects of colloids, especially hydroxyethyl starches for fluid resuscitation in sepsis [32, 62, 128]. However, the risk of acute kidney injury requiring renal replacement therapy is substantially increased by the use of hydroxyethyl starch (EI) [128].

While a large randomized study indicated that albumin administration was safe and equally effective as 0.9 % saline [50], a meta-analysis of data from 17 randomized trials found that the use of albumin-containing solutions for fluid resuscitation of patients with sepsis was associated with lower mortality compared with crystalloids [40]. However, in a multicenter randomized trial (*n* = 794) in patients with septic shock, the use of albumin therapy did not significantly reduce 28-day mortality compared to saline solution [50]. Thus, albumin-containing solutions may be used for fluid resuscitation of patients with sepsis and septic shock (CII).

If a sufficient mean arterial pressure (>65 mmHg) cannot be achieved by volume substitution in a reasonable time frame, treatment with vasopressors is indicated. The drug of choice to elevate the vasotonus is norepinephrine in a dose of 0.1–1.3 μg/kg/min (BII) [48].

In retrospective and small prospective studies, vasopressin (0.01–0.04 U/min) increased urinary output and creatinine clearance compared to norepinephrine [51–53]. However, in the large VASST trial, no reduction in 28-day mortality was found in the vasopressin group, and there is currently poor evidence to support the use of vasopressin in septic shock (CI) [54]. In case of sepsis-related myocardial depression leading to low cardiac output despite adequate volume substitution, vasopressor treatment with dobutamine should be instituted (AII) [140]. Of note, results from an observational study suggest that dopamine administration may be associated with increased mortality rates in septic patients [142].

Bicarbonate therapy is not recommended for the purpose of improving haemodynamics or reducing vasopressor requirements in the presence of lactic acidosis and pH >7.15 (DII) [38, 108].

### Treatment of pulmonary failure

Pneumonia leading to acute respiratory failure is a major cause of sepsis in neutropenic cancer patients [19, 20]. On the other hand, severe sepsis may lead to acute lung injury/acute respiratory distress syndrome (ARDS) [145].

In cooperative and awake patients with mild to moderate pulmonary failure, non-invasive positive pressure ventilation should be preferred (AII) [19, 37, 69, 70, 145]. Both non-invasive treatment options led to a significant reduction of intubation compared to the control group in neutropenic cancer patients [19, 73]. An early start of non-invasive ventilation, prior to development of severe hypoxaemia, is favourable (BII) [21]. Failure of non-invasive ventilation occurs in half of the critically ill haematologic patients and is associated with an increased mortality [21]. Predictors of non-invasive ventilation failure are as follows: high respiratory rate, short time between admission and non-invasive ventilation [21], vasopressor use, renal replacement therapy and the development of ARDS [3].

In moderate to severe respiratory insufficiency, endotracheal intubation and mechanical ventilation are necessary. Survival is positively correlated to the experience of the ICU with haematologic and oncologic patients [171]. In a retrospective multicenter study of allogeneic HSCT recipients admitted to the ICU, mechanical ventilation was associated with low survival rates [107]. These data were confirmed in a prospective study [19].

Percutaneous extracorporeal membrane oxygenation showed to be a rescue therapy to bridge hypoxemia due to ARDS in patients with oncological or malignant haematological diseases [59, 99, 111]. Further studies are needed in this field, before a recommendation can be given.

### Management of renal dysfunction

Acute kidney injury (AKI) develops in approximately 20 % of patients with severe sepsis and 50 % with septic shock. The combination of acute renal failure and sepsis is associated with a 70 % mortality [144]. Specific data for neutropenic patients are lacking, and recommended management of renal dysfunction is not different from non-neutropenic patients.

In short, no clear guidelines on the timing of the initiation of renal replacement therapy (RRT) can be given. Regarding the mode of replacement therapy, intermittent haemodialysis and continuous renal replacement therapies (CRRT) are equivalent in patients with sepsis and AKI (BI) [57, 58, 84, 100, 134, 155]. In haemodynamically unstable patients, control of fluid balance may be facilitated by the use of CRRT (BII) [42, 79].

Increasing the frequency of RRT is thought to reduce the rate of uremic complications and improve outcome in patients with AKI. However, randomized controlled studies showed conflicting results [42, 79, 138]. A recent meta-analysis indicates that high-dose RRT in critically ill patients with AKI does not improve patient survival or recovery of renal function as compared with less-intensive regimes [75, 122, 160]. Thus, no firm recommendations can be given for the increased frequency of RRT (CI).

In patients undergoing renal replacement therapy, the dosage of antimicrobial substances should be carefully checked and adjusted [86]. The use of low-dose dopamine for protection of renal function is not recommended (EI) [26, 85].

### Nutrition and control of metabolic functions

#### Caloric intake

Most recommendations reported are extrapolated from analyses in critically ill and well-nourished patients without neutropenia. Enteral nutrition is preferred over parenteral nutrition unless contraindicated or impossible, as it is associated with a lower rate of infections (BII) [67]. Enteral caloric intake should be calculated according to the phase of sepsis: during the initial phase of sepsis, the supply of >20–25 kcal/kg ideal bodyweight (IBW) has been associated with inferior outcome in one observational study (DIII) [91]. During recovery, 25–30 kcal/kg IBW should be provided (BIII) [89, 149].

#### Supplements

As reproducible mortality benefits for supplementation of arginine [31, 56], omega-3 fatty acids [24, 130, 131] and combined formulations [27, 56, 68] in patients with severe sepsis and septic shock are lacking, we do not recommend general use of either of these supplements (DII). Substitution of glutamine did not positively affect the primary survival endpoint in two randomized trials including together over 1,000 patients with sepsis [9, 66] and significantly increased in-hospital and 6-month mortality in the REDOXS study [66]. Therefore, glutamine substitution cannot be recommended (EI).

Two recent large randomized two-factorial trials compared the influence of lower doses of selenium substitution to placebo in critically ill patients [9, 66]. In these trials including 282 and 826 septic patients, respectively, selenium substitution had no effect on mortality [9, 66]. In a smaller trial, higher dose selenium substitution (1,000 μg daily) was associated with mortality reduction only in the per protocol analysis [10]. In a meta-analysis including nine trials with septic patients, selenium substitution was associated with lower mortality, especially in patients receiving selenium in higher doses (≥1,000 mg daily) and for ≥6 days [74]. Thus, further clinical trials regarding the adequate dosing and treatment duration are needed before treatment with selenium can be recommended (DI).

#### Hyperglycaemia

Hyperglycaemia in patients requiring intensive care is associated with an inferior outcome [23, 47]. Results of clinical trials in patients with severe sepsis and septic shock [13, 32] as well as clinical trials [51, 133, 158] including patients with underlying malignant disease [158] and a meta-analysis [61] in mixed populations of critically ill patients failed to show a benefit of intensified insulin therapy. These results are in contrast to the results of the initial trial by van den Berghe et al. [159] which had suggested a benefit of a tight blood glucose control (blood glucose level of 4.4–6.6 mmol/L (80–120 mg/dL)). Thus, we do not recommend intensive insulin therapy aiming at a blood glucose level of 4.4–6.6 mmol/L (80–120 mg/dL; EI). Based on these data and international guidelines [44, 76, 137], we recommend to maintain blood glucose levels at least ≤9.9 mmol/L (≤180 mg/dL; BIII). A high variability of blood glucose levels in septic patients should be avoided, as this is associated with increased mortality (BIII) [7, 22, 46].

### Treatment with corticosteroids

Replacement of an impaired adrenal reserve and anti-inflammatory properties is a rationale for studying corticosteroids as an adjunctive to sepsis therapy. The use of corticosteroids in sepsis has not been studied in a prospective fashion for neutropenic patients. In the CORTICUS trial, 84 cancer patients were included; however, no subgroup analysis of these patients has been published [35].

#### High-dose corticosteroid treatment (>300 mg hydrocortisone per day)

Randomized controlled trials and meta-analyses reported on increased overall mortality and increased mortality from secondary infections in non-neutropenic patients with sepsis receiving high-dose steroids [28, 39, 113, 153, 163]. Thus, high-dose corticosteroids are not recommended as treatment of sepsis (EI).

#### Low-dose corticosteroid treatment (≤300 mg hydrocortisone per day)

Substitutive doses of hydrocortisone during sepsis remain controversial. Annane et al. identified a benefit in 28-day mortality for the treatment group [14]. The CORTICUS trial did not reveal a difference in 28-day mortality between treatment and placebo and found a higher incidence of hyperglycaemia, hypernatraemia and secondary infections in the treatment group [152]. The results of meta-analyses have been similarly contradictory. Some meta-analyses support the use of low doses of hydrocortisone [12, 114, 150], while others do not support the use of low doses of hydrocortisone [80, 116, 146]. Newer data from three observational studies with a total of over 25,000 patients from sepsis registries showed no mortality benefit for low-doses steroids [25, 33, 49]. Thus, we do not recommend the routine use of substitutive doses of hydrocortisone in neutropenic patients with sepsis (DI). However, low-dose corticoid treatment may be considered in patients with insufficient restoration of blood pressure levels despite adequate fluid resuscitation and vasopressor treatment (BII) [124]. The results of three ongoing large randomized controlled trials will hopefully further clarify the role of low-dose steroids in severe sepsis.

### Treatment with coagulation inhibitors

In sepsis, the coagulation cascade is frequently activated at early time points. As thrombocytopenia and an increased risk of bleeding are frequently present in patients with cancer and chemotherapy, attempts to positively influence coagulation in patients with neutropenia have to be exerted carefully.

#### Heparin

Retrospective trials in patients with sepsis have shown a reduction in mortality using unfractionated heparin [170]. The prospective randomized controlled HETRASE study has investigated treatment with low-dose heparin (500 IU/h during 7 days) in 319 patients with sepsis [77]. No influence on 28-day all-cause mortality was found. This trial was characterized by low mortality, perhaps explained by liberal inclusion criteria. Treatment was discontinued when the partial thromboplastin time exceeded 60 s. Under these conditions, the administration of low-dose heparin was safe. Further trials including more patients and defined subgroups are needed before recommendations for the use of heparin in neutropenic sepsis can be made (CI).

#### Antithrombin

Antithrombin has anti-thrombotic and anti-inflammatory properties. Based on the negative data from the KyberSept trial [165], a Cochrane analysis [4], and subgroup analyses of several trials [72, 87, 167], we do not recommend the routine use of antithrombin as treatment of neutropenic sepsis in the absence of disseminated intravascular coagulation (DIC; DI). However, in patients with DIC and sepsis, the administration of antithrombin may be considered (BII) [87].

#### Activated protein C (APC)

In response to the results of the PROWESS-SHOCK trial [136], APC is no longer in use.

#### Thrombomodulin

Thrombomodulin decreases thrombus formation, activates protein C and has anti-inflammatory properties [2, 119, 148, 161]. Results from a phase IIb study suggested efficacy in patients with sepsis and suspected DIC [161]. However, safety and efficacy is not known in cytopenic patients, and no evidence-based recommendation can be made.

### Cytokines and haematopoietic growth factors (G-CSF, GM-CSF)

The central role of cytokines during the hyper- and anti-inflammatory phases of sepsis prompted clinical studies on the use of cytokines and cytokine inhibitors as therapeutic agents. However, studies on the therapeutic efficacy of IL-1 receptor antagonist, TNF-inhibitors, TLR-4 inhibitors and interferon gamma did not show a clinical benefit (EI) [52, 53, 117, 121, 139].

The known effect of G-CSF and GM-CSF in increasing the number of circulating granulocytes was the rationale for clinical studies assessing their role as additional therapy to antibiotics in febrile patients with chemotherapy-induced neutropenia. A meta-analysis of 13 randomized controlled trials including a total of 1,518 patients showed that G-CSF/GM-CSF effectively reduces the time to neutrophil recovery and the length of hospitalization [36]. However, despite a marginally significant benefit for the use of G-CSF/GM-CSF in reducing infection-related mortality, overall mortality appeared not to be influenced. Even though this meta-analysis reported only mild side effects associated with G-CSF/GM-CSF treatment (bone pain, joint pain and flue-like symptoms), there is an accumulating number of publications on respiratory deterioration with ARDS during G-CSF/GM-CSF-induced neutropenia recovery [17, 18, 83, 147]. In non-neutropenic patients with pneumonia or sepsis, G-CSF/GM-CSF appeared to be safe but ineffective in reducing mortality rates or complications from infection [141, 169]. On the basis of the current studies and reports, we do not recommend the routine additional use of G-CSF or GM-CSF to standard treatment of sepsis in neutropenia (DI). Although GM-CSF seems to be able to reverse sepsis-induced immune paralysis, it is currently not available for treatment in the EU [110, 143].

### Immunoglobulins

The treatment of sepsis in neutropenia with i.v. immunoglobulin’s (IVIG) did not show a significant difference in survival in a randomized controlled trial [65]. A meta-analysis on trials of IVIG in patients with sepsis identified 20 trials eligible for evaluation [156]. Compared with placebo or no intervention, the use of polyclonal IVIG was associated with a survival benefit (relative risk 0.74). The number needed to treat to save one life was nine. Interestingly, more severely ill patients, those receiving treatment for more than 2 days and those receiving ≥1 g/kg, seemed to benefit most. As most of the individual trials analyzed had flaws in design, were rather small or performed during a time when the standard of care for septic patients was different from today; the authors conclude that a large randomized controlled trial should be performed [156]. Three additional meta-analyses investigated the use of IVIG during sepsis and had similar outcomes [90, 94, 151]. In conclusion, there is moderate degree of evidence to support the use of IVIG in sepsis (BII).

### Granulocyte transfusions

Several case reports and phase I/II studies have shown some efficacy of granulocyte transfusions in patients with infections during severe neutropenia including patients with invasive fungal infections. However, complications have been reported as well, e.g. fatal CMV infection, allo-immunization and the transfusion-related acute lung injury syndrome. Recently, a randomized controlled trial has been published [101]. It failed to show any beneficial effect, but it was small, and the authors discussed several problems associated with the design of the trial. A meta-analysis of the use of granulocyte transfusion in neutropenic neonates yielded equivocal results [123]. Taken together, at this time, no recommendation can be given on the use of granulocyte transfusions outside of clinical trials.

### Transfusion management in sepsis

The recommendations for substituting platelets or packed red blood cell in neutropenic patients can be applied to those patients developing sepsis as well. However, the cutoff for substitution is often set to higher values (platelets 20,000/μL instead of 10,000/μL; BIII). A randomized trial in children with sepsis showed no difference in outcome between a liberal (<9.5 g/dL) and a restrictive (<7 g/dL) trigger for erythrocyte transfusion [82]. Although there are no prospective randomized studies available, we recommend a transfusion trigger of <9 g/dL haemoglobin level to optimize tissue oxygenation (there was no consensus inside the panel of experts regarding the strength of recommendation) [166].

## Summary of recommendations

Table 5 summarizes treatment recommendations given by the AGIHO.

**Table 5:** Summary of treatment recommendations given by the Infectious Diseases Working Party of the German Society of Hematology and Medical Oncology (AGIHO)

Recommendation	Evidence level
Antimicrobial treatment	
Initial treatment with meropenem or with imipenem/cilastatin or with piperacillin/tazobactam	AIII
A combination treatment with an aminoglycoside in neutropenic patients with septic shock and severe sepsis may be considered	BIII
Cardiovascular insufficiency	
Albumin-containing solutions may be used in patients with sepsis and septic shock	CII
The drug of choice to elevate the vasotonus is norepinephrine	BII
In case of sepsis-related myocardial depression leading to low cardiac output despite adequate volume substitution, vasopressor treatment with dobutamine should be instituted	AII
Treatment of pulmonary failure	
Non-invasive positive pressure ventilation (CPAP or bilevel positive airway pressure) should be preferred if possible in patients without hypotension or altered mental status	AII
An early start of non-invasive ventilation, prior to development of severe hypoxaemia, is favourable	BII
Management of renal dysfunction	
Intermittent haemodialysis and continuous renal replacement therapies are equivalent	BI
No firm recommendations can be given for the use of increased doses of renal replacement therapy	CI
Low-dose dopamine for protection of renal function is not recommended	EI
Nutrition and control of metabolic functions	
Enteral nutrition is preferred over parenteral nutrition	BII
During initial phase of sepsis, energy supply should not exceed 20–25 kcal/kg ideal bodyweight (IBW)	DIII
During recovery, 25–30 kcal/kg IBW should be provided	BIII
We do not recommend general use of arginine, omega-3 fatty acids and combined formulations in patients with severe sepsis and septic shock	DII
Glutamine substitution cannot be recommended in patients with severe sepsis and septic shock	EI
Further clinical trials regarding the adequate dosing and treatment duration are needed before treatment with selenium can be recommended	DI
Aiming at strictly normal blood glucose level of 4.4–6.6 mmol/L (80–120 mg/dL) is not recommended	EI
Blood glucose levels should be kept ≤9.9 mmol/L (≤180 mg/dL) in septic neutropenic patients	BIII
A high variability of blood glucose levels in septic patients should be avoided, as this is associated with increased mortality	BIII
Corticosteroids	
High-dose corticosteroids should not be used in neutropenic or non-neutropenic septic patients	EI
The routine use of substitutive doses of hydrocortisone in neutropenic patients with sepsis is not recommended	DI
Low-dose corticoid treatment may be considered in patients with insufficient restoration of blood pressure levels despite adequate fluid resuscitation and vasopressor treatment	BII
Treatment with coagulation inhibitors	
Further trials on the use of low-dose heparin (500 IU/h for 7 days) are needed before recommendations can be made	CI
Routine use of antithrombin is not recommended as treatment of sepsis in neutropenic patients antithrombin may be considered during DIC and sepsis	DI
	BII
Growth factors and immunoglobulins	
The routine additional use of G-CSF or GM-CSF as standard treatment of sepsis in neutropenia is not recommended	DI
There is moderate degree of evidence to support the use of i.v. immunoglobulins in sepsis	BII
Transfusion management	
The cut-off for substitution of platelets is often set to a higher value (platelets 20,000/μL instead of 10,000/μL during sepsis)	BIII
A transfusion trigger of <9 g/dL haemoglobin during neutropenic sepsis is recommended	BIII
